# B-cell receptor signaling induces proteasomal degradation of PDCD4 via MEK1/2 and mTORC1 in malignant B cells

**DOI:** 10.1016/j.cellsig.2022.110311

**Published:** 2022-06

**Authors:** Joe Taylor, Sarah Wilmore, Sophie Marriot, Karly-Rai Rogers-Broadway, Rachel Fell, Annabel R. Minton, Tom Branch, Meg Ashton-Key, Mark Coldwell, Freda K. Stevenson, Francesco Forconi, Andrew J. Steele, Graham Packham, Alison Yeomans

**Affiliations:** a Cancer Research UK Centre, Cancer Sciences, Faculty of Medicine, University of Southampton, Southampton, United Kingdom; b Department of Cellular Pathology, Southampton General Hospital, Southampton, United Kingdom; c Centre for Biological Sciences, Faculty of Natural and Environmental Sciences, University of Southampton, Southampton, United Kingdom

**Keywords:** Chronic lymphocytic leukemia, Lymphoma, PDCD4, mRNA translation, Ibrutinib, eIF, eukaryotic initiation factor, BCR, B-cell receptor, CLL, chronic lymphocytic leukemia, DLBCL, diffuse large B-cell lymphoma, BL, Burkitt's lymphoma, CHX, cycloheximide, DMSO, dimethylsulfoxide, sIgM, surface IgM, PC, proliferation center, PSI, proteasome inhibitor, GCB, germinal center B-cell, ABC, activated B-cell

## Abstract

B-cell receptor (BCR) signaling plays a major role in the pathogenesis of B-cell malignancies and is an established target for therapy, including in chronic lymphocytic leukemia cells (CLL), the most common B-cell malignancy. We previously demonstrated that activation of BCR signaling in primary CLL cells downregulated expression of PDCD4, an inhibitor of the translational initiation factor eIF4A and a potential tumor suppressor in lymphoma. Regulation of the PDCD4/eIF4A axis appeared to be important for expression of the MYC oncoprotein as *MYC* mRNA translation was increased following BCR stimulation and MYC protein induction was repressed by pharmacological inhibition of eIF4A. Here we show that MYC expression is also associated with PDCD4 down-regulation in CLL cells in vivo and characterize the signaling pathways that mediate BCR-induced PDCD4 down-regulation in CLL and lymphoma cells. PDCD4 downregulation was mediated by proteasomal degradation as it was inhibited by proteasome inhibitors in both primary CLL cells and B-lymphoma cell lines. In lymphoma cells, PDCD4 degradation was predominantly dependent on signaling via the AKT pathway. By contrast, in CLL cells, both ERK and AKT pathways contributed to PDCD4 down-regulation and dual inhibition using ibrutinib with either MEK1/2 or mTORC1 inhibition was required to fully reverse PDCD4 down-regulation. Consistent with this, dual inhibition of BTK with MEK1/2 or mTORC1 resulted in the strongest inhibition of BCR-induced MYC expression. This study provides important new insight into the regulation of mRNA translation in B-cell malignancies and a rationale for combinations of kinase inhibitors to target translation control and MYC expression.

## Highlights

• B-cell receptor signaling induces rapid PDCD4 downregulation in malignant B cells.
• PDCD4 downregulation is dependent on increased proteasomal degradation.
• PDCD4 downregulation is mediated via the mTORC1/p70-S6K and ERK1/2 pathways.
• PDCD4 downregulation correlates with increased MYC expression in vitro and in vivo.

## 1 Introduction

Programmed cell death protein 4 (PDCD4) is a multi-functional regulator of apoptosis, cell growth and transformation which acts predominantly via inhibition of eukaryotic initiation factor (eIF) 4A, a core component of the eIF4F translation initiation complex [1], [2], [3]. The RNA helicase activity of eIF4A is required for translation of mRNAs with highly structured 5′-untranslated regions and PDCD4 is therefore thought to preferentially suppress translation of a subset of mRNAs, including those encoding major oncoproteins such as MYC [4], [5]. PDCD4 acts as a tumor suppressor as its expression is frequently downregulated in cancers compared to normal tissues [6], [7], [8] and *Pdcd4* deletion is associated with increased lymphomagenesis in mice [9]. Multiple mechanisms can contribute to PDCD4 expression down-regulation, including reduced transcription [10], [11] and miRNA-mediated repression [12], [13]. PDCD4 can also be targeted for degradation via the proteasome by signaling through the ERK/p90RSK or AKT/mTORC1/p70S6K pathways [14], [15], [16], [17], [18], [19], [20], [21].

We previously investigated regulation of mRNA translation following activation of the cell surface B-cell receptor (BCR) in chronic lymphocytic leukemia (CLL), the most common mature B-cell malignancy [22], [23]. BCR signaling is a major driver of cell survival and proliferation in CLL and other B-cell malignancies, including diffuse large B-cell lymphoma (DLBCL) and Burkitt's lymphoma (BL) [24], [25], [26]. The mechanisms that drive BCR signaling may differ between these malignancies [27]. In CLL, signaling appears to be triggered by engagement of the BCR by (auto)antigen, although BCR-BCR interactions may also play a role. Consistent with this, inhibitors targeted against BCR signaling-associated kinases, such as the BTK inhibitor ibrutinib, have revolutionized treatment of CLL [28]. In DLBCL cell lines, cell survival signaling can be driven by engagement of the BCR by self-antigen (i.e., antigen expressed by the lymphoma cells) or by a low-level, antigen-independent signal emanating from the BCR [29], [30], [31] similar to antigen-independent “tonic” signaling originally described in normal B cells [32], [33].

In our previous study of CLL cells, we showed that stimulation of primary CLL cells using anti-IgM to mimic engagement of the BCR by antigen increased global mRNA translation, as well as translation of the *MYC* mRNA [23]. This induction of mRNA translation was associated with down-regulation of PDCD4 (as well as increased expression of the eIF4F components, eIF4A and eIF4E). Modulation of the PDCD4/eIF4A axis appeared to be critical for the induction of MYC, since eIF4A inhibitors effectively reduced anti-IgM-induced MYC expression [34], [35]. Given the role of PDCD4 as suppressor of B-cell lymphomagenesis [9], and the importance of the PDCD4/eIF4A axis in control of MYC expression downstream of the BCR [34], here we have investigated the regulatory pathways that mediate PDCD4 down-regulation following BCR stimulation in malignant B cells.

## 2 Methods and methods

### 2.1 Reagents

Cycloheximide (CHX) and Q-VD-OPh were from Sigma (Poole, UK) and ipatasertib, rapamycin, LY2584702, U0126, ibrutinib, tamatinib, bortezomib and MG132 were from SelleckChem (Houston, US). All compounds were dissolved in dimethylsulfoxide (DMSO).

### 2.2 Patient samples and cells

The study received ethical approval (South Central - Hampshire B Research Ethics Committee), was performed in accordance with the Declaration of Helsinki of 1975 and all patients provided written informed consent. Peripheral blood mononuclear cells were collected from CLL patients and cryopreserved and recovered as described [36]. None of the patients received any (immuno)chemotherapy, steroids or ibrutinib for the 6 months prior to sample collection. Tumor *IGHV* mutational status and surface IgM (sIgM) expression/signaling capacity was analyzed as described [36]. The median proportion of CD5^+^CD19^+^ cells was 96% (range 67–99%) and all samples were considered as anti-IgM-responsive using a cut-off of anti-IgM-induced iCa^2+^ flux in ≥5% of cells. Cell viability determined by trypan blue exclusion after recovery was ≥90% in all cases. RL and RAMOS cells (DSMZ, Braunschweig, Germany) and HBL1 cells (a kind gift of Prof. Martin Dyer, University of Leicester, UK) were cultured in RPMI-1640 media supplemented with 10% fetal bovine serum, 2 mM l-glutamine and 1% penicillin/streptomycin for a maximum of 8 weeks. Cell line identity was routinely confirmed using short tandem repeat analysis (Powerplex 16 System, Promega, Southampton, UK) and absence of mycoplasma was confirmed using the Mycoplasma PCR detection kit (Applied Biological Materials, Richmond, Canada). sIgM stimulation was performed using either soluble (cell lines) or bead-bound (CLL samples) goat F(ab’)_2_ anti-human IgM (Cambridge Biosciences, Cambridge, UK), as described [37], [38]. For experiments involving incubations of CLL samples for ≥6 h, cells were treated with the caspase inhibitor Q-VD-OPh (5 μM) to minimize secondary events due to apoptosis.

### 2.3 Immunohistochemistry (IHC)

CLL LN sections (*n* = 6) were stained using a Leica Bond RX autostainer and detection reagents from Leica Biosystems (Wetzlar, Germany). All incubations were performed at room temperature and heat-induced epitope retrieval was performed by incubation in Bond epitope retrieval solution 2 for 20 min. Prior to antibody staining, hydrogen peroxide was added to the slides and incubated for 5 min, followed by three washes in Bond wash solution. The slides were then incubated with rabbit anti-PDCD4 antibody (Cell Signaling Technologies, 1:200 dilution for 30 min), biotinylated anti-rabbit antibody (8 min) and then streptavidin-enzyme polymer (8 min). Slides were washed between incubations in Bond wash solution, and finally with deionized water. 3,3′-diaminobenzidine was added and slides were incubated for 10 min before washes in deionized water (4 washes), Bond wash solution (1 wash) and deionized water (1 wash). The slides were then incubated with mouse anti-MYC (clone ab32072, 1:500 dilution for 30 min; Abcam, Cambridge, UK), post-primary IgG linker (20 min) and then alkaline phosphatase polymer (30 min). Slides were washed between incubations in Bond wash solution, and finally, with deionized water. Mixed Red Refine solution was added and slides were incubated for 10 min. Slides were washed and again incubated in mixed Red Refine solution for a further 5 min, prior to 3 washes in deionized water. Slides were counterstained with hematoxylin, followed by washing in deionized water, Bond wash solution and then deionized water. MYC and PDCD4 expression was counted in one field (~300 cells) from each of three proliferation centers (PC) in each LN (18 fields in total). Cell counts were recorded using CaseViewer Version 2.2 (3D Histech Ltd., Budapest, Hungary) and PCs were identified by parallel analysis of Ki67 stained sections.

### 2.4 Immunoblotting

SDS-PAGE was performed using equal protein loading following quantitation of protein content using the BioRad Protein Assay (BioRad, Hemel Hempstead, UK). Immunoblot analysis was performed using the following antibodies; anti-PDCD4 (Cell Signaling Technology, Danvers, US), anti-MYC (9E10; Merck Life Science UK, Gillingham, UK), anti-ubiquitin (P4D1; Santa Cruz Biotechnology, Dallas, US), anti-ERK1/2, anti–T^202^/Y^204^-phosphorylated ERK1/2, anti-p90RSK, anti-S^380^-phosphorylated p90RSK, anti-p70S6K, anti-T^389^-phosphorylated p70S6K, (all from Cell Signaling Technology), anti-β-actin (Cell Signaling Technology) and anti-HSC70 (Santa Cruz Biotechnology). Secondary antibodies were horseradish peroxidase-conjugated rabbit or mouse antibodies (GE Healthcare, Amersham, UK) and images were captured using the ChemiDoc-It Imaging System with a BioChemi HR camera (UVP, Cambridge, UK). Immunoblot signals were quantified using ImageJ (http://imagej.nih.gov/ij/). Expression of phospho-proteins were normalized to their corresponding total protein expression, whereas expression of all other proteins were normalized to a loading control.

### 2.5 Quantitative-polymerase chain reaction (Q-PCR)

Total mRNA were isolated by phenol/chloroform isolation. cDNA synthesis was performed using MMLV reverse transcriptase and oligo-dT primers (both Promega, Southampton, UK). *MYC, PDCD4* and *B2M* mRNA expression was quantified by Q-PCR using probes Hs00153408_m1, Hs00377253_m1 and Hs00984230_m1, respectively (Life Technologies, Carlsbad, US). mRNA abundance was determined for each mRNA against a standard curve, providing cDNA values and relative mRNA expression was calculated by normalizing the obtained values against *B2M* mRNA.

### 2.6 Statistics

Statistical comparisons were performed in Prism9 (GraphPad Software, La Jolla, USA) using Student's *t*-tests/one-sample t-tests or Wilcoxon tests/Wilcoxon signed-rank tests (depending on whether data were normally distributed or not, respectively, according to Shapiro-Wilk's tests), or Fisher's exact test.

## 3 Results

### 3.1 Reciprocal regulation of PDCD4 and MYC in vivo

**Fig. 1:**
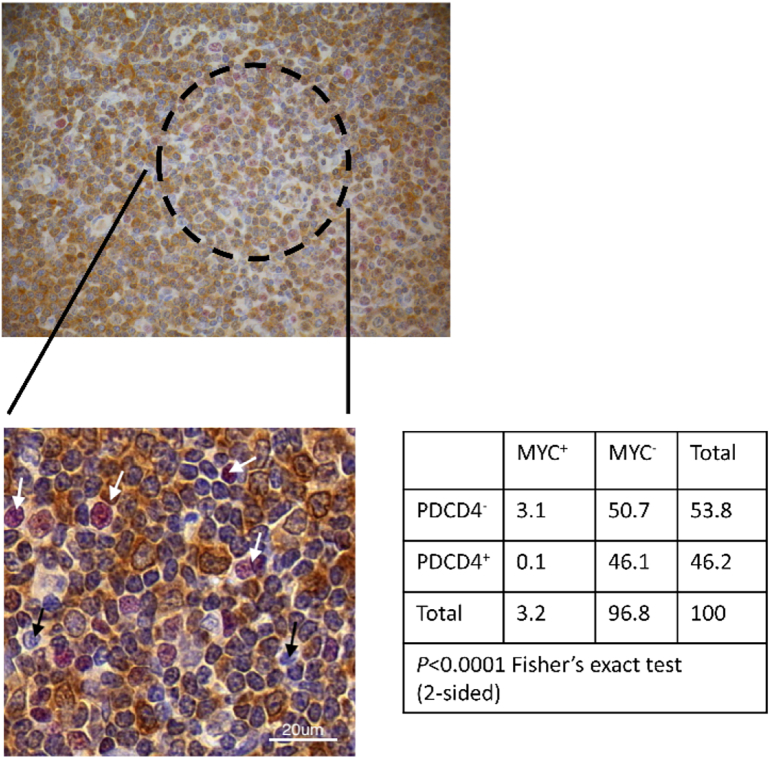
Reciprocal expression of MYC and PDCD4 in vivo. Expression of MYC (red) and PDCD4 (brown) was analyzed by dual-color IHC of CLL/small lymphocytic leukemia lymph node sections. Fig. shows IHC staining of a lymph node with a higher magnification image of a proliferation center (indicated by black dotted line). White and black arrows indicate MYC^+^/PDCD4^−^ and MYC^−^/PDCD4^−^ cells, respectively. The inset shows the percentage of MYC^+^/PDCD4^−^, MYC^+^/PDCD4^+^, MYC^−^/PDCD4^−^, MYC^−^/PDCD4^+^ cells (from a total of 5592 cells analyzed from 3 proliferation centers from 6 lymph nodes) and results of statistical analysis (Fisher's exact test). (For interpretation of the references to color in this figure legend, the reader is referred to the web version of this article.)

We previously showed that stimulation of the BCR of CLL cells resulted in up-regulation of MYC expression and down-regulation of PDCD4 expression. Moreover, inhibition of eIF4A (the target for PDCD4) effectively inhibits anti-IgM-induced MYC expression [34]. To determine whether the inverse relationship between MYC and PDCD4 expression is also observed in vivo we performed dual-color IHC of LN from patients with CLL/small lymphocytic lymphoma (Fig. 1). We focused on expression within proliferation centers (PC) which are the main sites of antigen engagement and proliferation for CLL cells in vivo [39].

As previously demonstrated [40], a small fraction of cells within the PC of each LN expressed MYC (mean 3.1%, range 0.4–7.1%) (Fig. 1). Whereas cells outside PCs were generally PDCD4 positive, a substantial proportion of PC cells lacked detectable PDCD4 expression (mean 51.2%, range 11.3–86.3%). Importantly, the vast majority (96%) of MYC positive cells within PCs were negative for PDCD4 and the inverse correlation between MYC and PDCD4 expression was highly significant. Therefore, PDCD4 expression is downregulated in a fraction of PC cells, and, similar to sIgM-stimulated CLL cells in vitro, this is associated with increased expression of MYC.

### 3.2 sIgM stimulation results in increased proteasomal degradation of PDCD4 protein

**Fig. 2:**
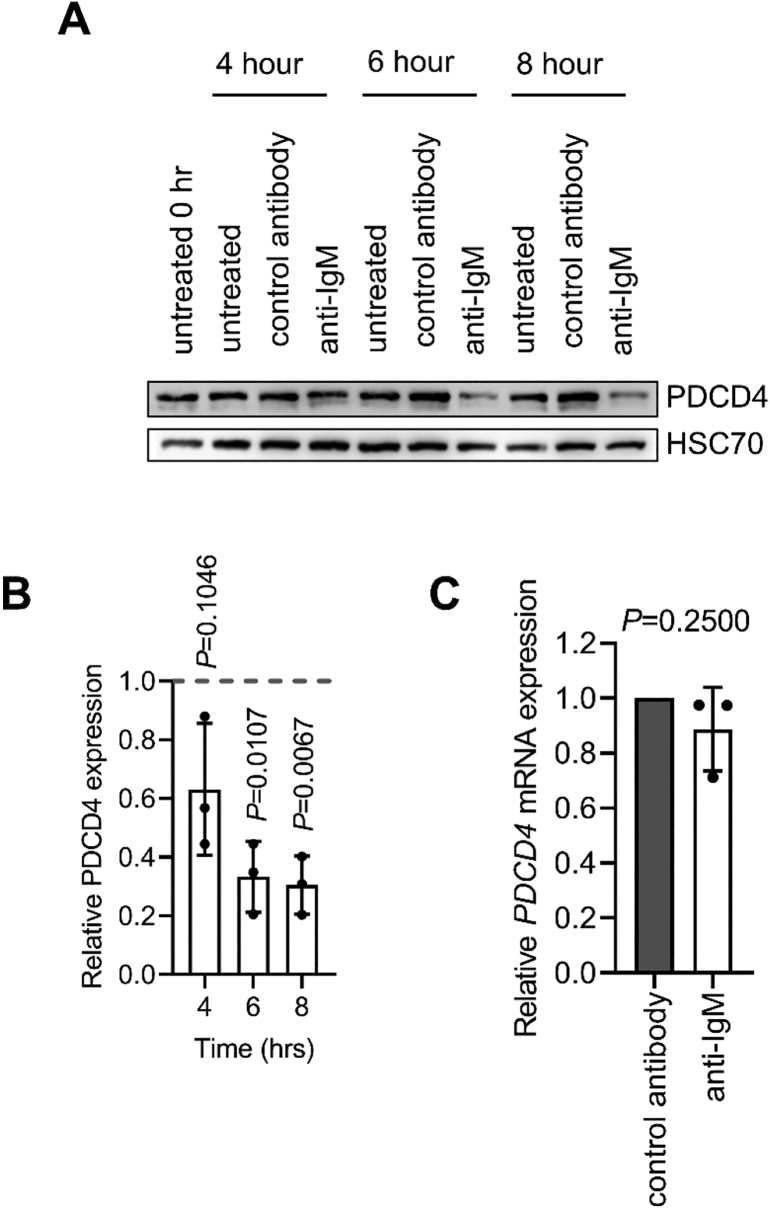
Regulation of PDCD4 by anti-IgM in CLL cells. *A*,*B*, CLL samples (*n* = 3) were treated with anti-IgM or control antibody beads, or left untreated for 0, 4, 6 or 8 h. *A*, representative immunoblot analysis of PDCD4 and HSC70 expression. *B*, quantification. *C*, CLL samples (*n* = 4) were treated with anti-IgM or control antibody beads for 6 h prior to analysis of *PDCD4* RNA expression by Q-PCR. All graphs show results for individual samples and mean (±SD) with values for control antibody-treated cells set to 1.0. The statistical significance compared to control antibody-treated cells is indicated (*B*, one sample *t*-tests; *C*,*D*, one-sample Wilcoxon tests).

Our previous study demonstrated that PDCD4 expression was downregulated at 24 h following stimulation of sIgM on primary CLL cells [23]. We therefore performed a time-course experiment to probe the kinetics of anti-IgM-induced PDCD4 down-regulation (Fig. 2A,B). The samples selected for study were all anti-IgM responsive [36] and comprised examples of both U-CLL and M-CLL, the two major subsets of CLL derived from pre- and post-germinal centre B-cell respectively (Supplementary Table 1). CLL cells express low levels of sIgM compared to normal B cells and we therefore used bead-bound anti-IgM (which induces stronger signaling compared to soluble anti-IgM) [38], [41] in these experiments. PDCD4 expression was reduced in all samples at 4 h although the degree of down-regulation varied between samples, consistent with the known variation of sIgM signaling strength in CLL cells [36]. PDCD4 expression was more strongly down-regulated at 6 and 8 h post-stimulation and at these times PDCD4 expression was significantly reduced compared to cells treated with control antibody. Therefore, anti-IgM-induced PDCD4 down-regulation is a relatively rapid response.

Previous studies have demonstrated that PDCD4 can be regulated at the level of transcription (e.g. by MYB) and translation (e.g. by miRNAs) [10], [11], [12], [13]. However, PDCD4 down-regulation following sIgM stimulation in CLL cells was not due to transcriptional regulation as Q-PCR analysis confirmed our previous finding, based on publicly available gene expression data [23], that down-regulation of PDCD4 protein by anti-IgM was not associated with a parallel reduction in *PDCD4* mRNA (Fig. 2C).

**Fig. 3:**
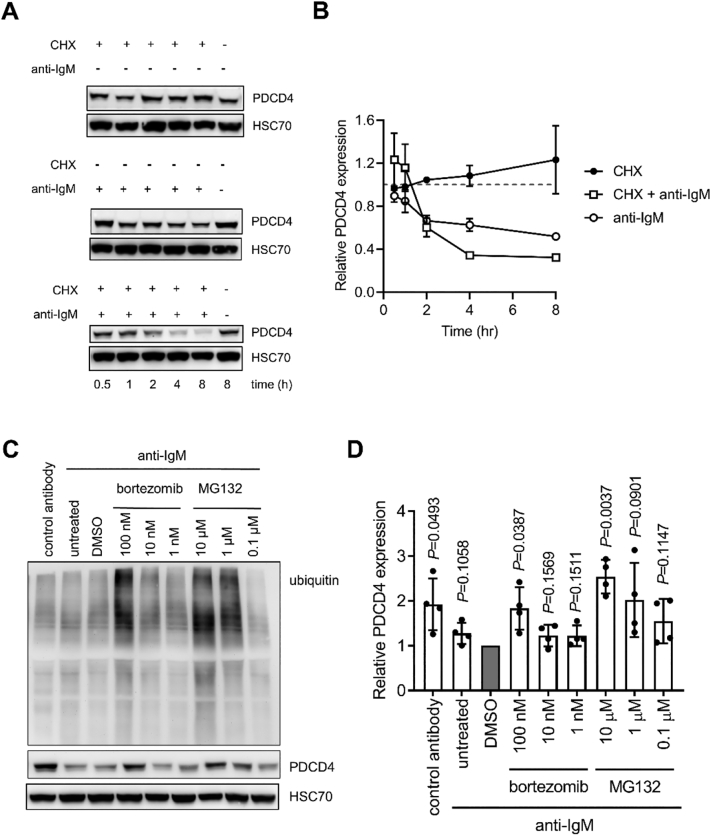
Analysis of PDCD4 stability and proteasomal degradation. *A*,*B,* CLL samples (*n* = 2) were pre-treated with CHX (30 μM) (or left untreated as a control) for 5 min and then incubated in the presence or absence of anti-IgM beads for the indicated times. PDCD4 and HSC70 expression was analyzed by immunoblotting. *A*, Representative immunoblots. *B*, quantification (mean ± range PDCD4 expression with values for 8 h/untreated samples set to 1.0). *C*,*D*, CLL samples (*n* = 4) were pre-treated with the indicated concentrations of bortezomib, MG132 or DMSO, or left untreated for 1 h, prior to treatment with anti-IgM or control antibody beads for 4 h. Expression of PDCD4, polyubiquitylated proteins and HSC70 was analyzed by immunoblotting. *C*, Representative immunoblots. *D*, quantification showing results for individual samples and mean (±SD) PDCD4 expression with values for anti-IgM/DMSO-treated cells set to 1.0. The statistical significance compared to control cells is indicated (one-sample *t*-tests).

To investigate potential post-translational regulation, we analyzed PDCD4 protein stability in the presence or absence of anti-IgM. CLL cells were treated with anti-IgM in the presence or absence of CHX to inhibit new protein synthesis (Fig. 3A). PDCD4 expression was essentially unchanged up to 8 h after addition of CHX in unstimulated CLL cells demonstrating that PDCD4 is relatively stable in the absence of sIgM stimulation and that the down-regulation of PDCD4 in anti-IgM-treated cells must involve accelerated turnover of the protein. Consistent with this, CHX accelerated down-modulation of PDCD4 in anti-IgM-treated cells consistent with the idea that PDCD4 proteolysis is increased under these conditions.

We investigated the role of the proteasome in anti-IgM-induced PDCD4 down-modulation using the proteasome inhibitors (PSI) MG132 and bortezomib. Because the extent of PDCD4 down-regulation by anti-IgM varied between samples, PDCD4 expression values were normalized to anti-IgM/DMSO-treated cells. PSI inhibited anti-IgM-induced PDCD4 and the effects of PSI on PDCD4 expression were mirrored by the accumulation of higher molecular weight polyubiquitylated proteins revealing a close link between proteasome inhibition and PDCD4 accumulation (Fig. 3C,D). Therefore, sIgM stimulation results in PDCD4 downregulation by increased proteasome-mediated turnover.

### 3.3 AKT or MEK pathway inhibition partially reverses anti-IgM-induced PDCD4 down-regulation in CLL cells

**Fig. 4:**
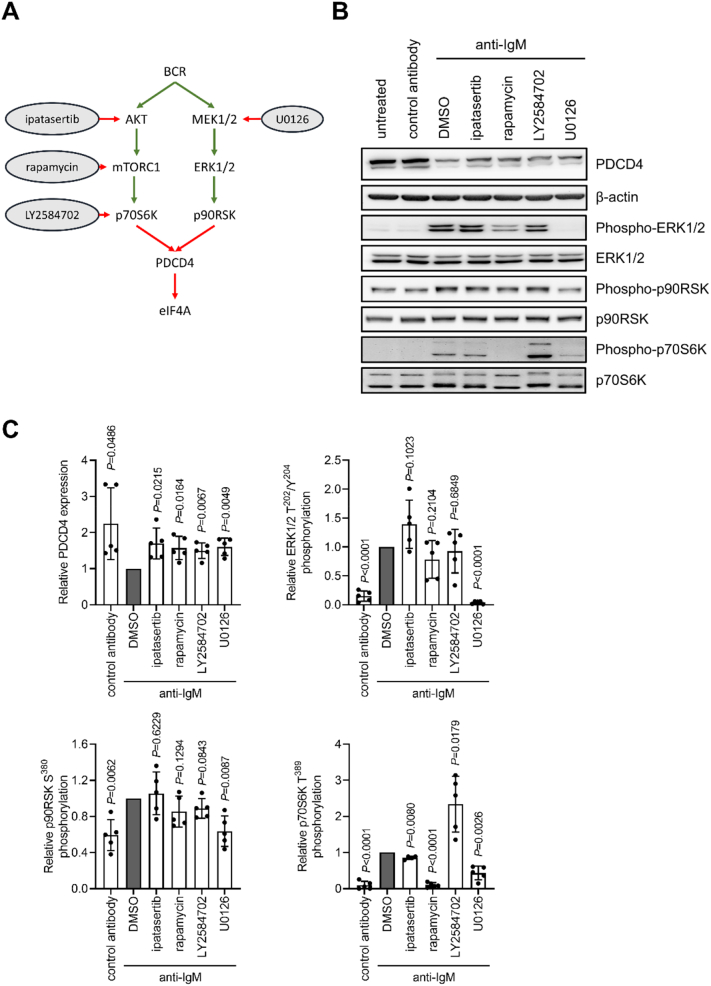
The effect of kinase inhibition on anti-IgM-induced PDCD4 down-regulation and signaling in CLL cells. *A*, BCR signaling pathways acting upstream of PDCD4 with inhibitors shown. Green and red arrows shown activating and inhibitory effects, respectively. *B*,*C*, CLL samples (*n* = 5) were pre-treated with ipatasertib, LY2584702, U0126, (all 10 μM), rapamycin (50 nM), DMSO, or left untreated for 1 h prior to treatment with anti-IgM or control antibody beads for 6 h. Expression of PDCD4, β-actin and total/phospho-p70S6K, ERK1/2 and p90RSK was analyzed by immunoblotting. *B*, Representative immunoblots. *C*, Quantification showing results for individual samples and mean (±SD) expression/phosphorylation with values for anti-IgM/DMSO-treated cells set to 1.0. The statistical significance compared to control cells is indicated (one sample *t*-tests). (For interpretation of the references to color in this figure legend, the reader is referred to the web version of this article.)

We next investigated the role of kinases activated downstream of the BCR in mediating anti-IgM-induced PDCD4 down-regulation (Fig. 4A). In other cell types, activation of the AKT/mTORC1 or ERK1/2 signaling pathways induce PDCD4 degradation [16], [17], [18], [19], [21] and we therefore investigated the effects of inhibitors of AKT, mTORC1 or p70S6K (ipatasertib, rapamycin or LY2584702, respectively) and MEK1/2 (U0126). As for analysis of PSI, the effect of inhibitors was analyzed following normalization to expression in anti-IgM/DMSO-treated cells.

The AKT pathway inhibitors and the MEK1/2 inhibitor U0126 significantly increased PDCD4 expression in anti-IgM-treated cells compared to DMSO-treated cells (Fig. 4B,C). However, when considering all samples together, none of these compounds were able to fully reverse the down-modulating effects of anti-IgM (all inhibitors reversed anti-IgM-induced down-modulation by ~60% (Fig. 4C)). The inhibitors appeared to more effectively reverse PDCD4 down-regulation in samples where PDCD4 downregulation by anti-IgM was modest and were less effective in samples with stronger anti-IgM-induced PDCD4 downregulation (Supplementary Fig. 1). Thus, the failure to fully reverse PDCD4 down-modulation appeared to be linked to the extent to which anti-IgM decreased PDCD4 expression. By contrast, inhibition of SYK (which initiates signaling downstream of the BCR and is required for activation of both the AKT/mTORC1 and ERK1/2 pathways) very effectively reversed anti-IgM-induced PDCD4 down-modulation (Supplementary Fig. 1).

We investigated the effects of the inhibitors on signaling, especially phosphorylation of p70S6K and p90RSK which are downstream targets of the AKT pathway and MEK1/2 and have been linked to PDCD4 phosphorylation [14], [15], [16], [17], [18], [19], [20], [21]. This demonstrated that, as expected, rapamycin (and to a lesser extent ipatasertib) significantly reduced downstream p70S6K phosphorylation but had no effect on the MEK pathway (ERK1/2 and p90RSK phosphorylation) (Fig. 4B,C). The MEK1/2 inhibitor U0126 significantly reduced ERK1/2 and p90RSK1 phosphorylation, although changes in p90RSK1 phosphorylation were generally modest as CLL cells appeared to have relatively high levels of basal phosphorylation in the absence of stimulation. Interestingly, U0126 also significantly reduced p70S6K phosphorylation, revealing cross-talk from the MEK to AKT pathways.

Overall, both the MEK and AKT pathways contribute to PDCD4 down-regulation in CLL cells following sIgM stimulation. However, blockade of either pathway failed to completely suppress PDCD4 down-regulation, especially in samples with the strongest degree of PDCD4 down-regulation.

### 3.4 AKT pathway inhibition fully reverses anti-IgM-induced PDCD4 down-regulation in B-lymphoma cells

**Fig. 5:**
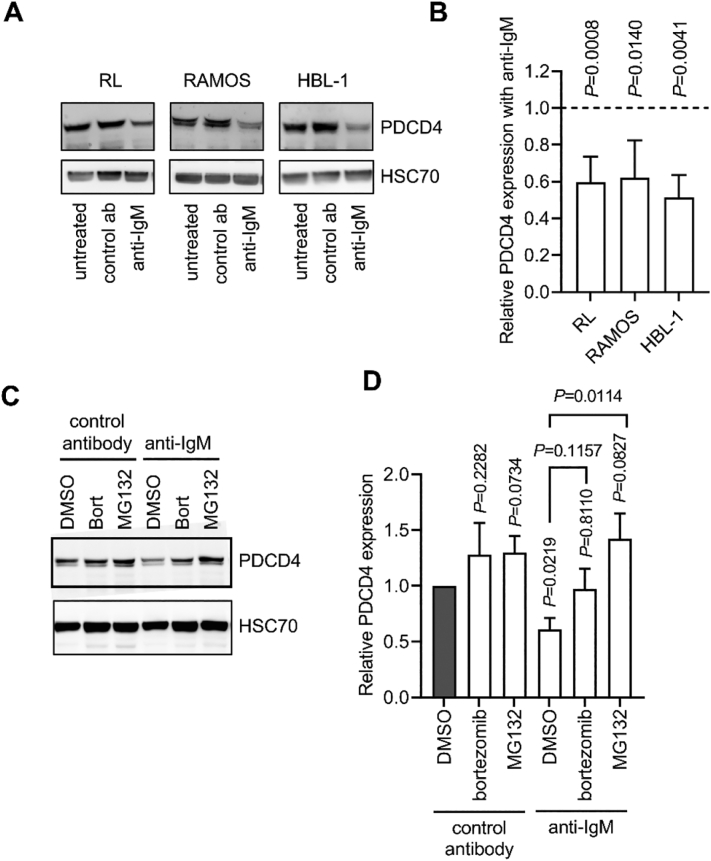
Proteasomal regulation of PDCD4 in anti-IgM stimulated B-cell lymphoma cell lines. *A*,*B*, RL, RAMOS and HBL-1 cells were treated with soluble control antibody or anti-IgM, or left untreated for 6 h. Expression of PDCD4 and HSC70 was analyzed by immunoblotting. *A*, Representative results. *B*, quantitation showing effect of anti-IgM on PDCD4 expression (mean ± SD) derived from 4 to 6 independent experiments with values for control antibody-treated cells set to 1.0. The statistical significance compared to control antibody-treated cells is indicated (one-sample *t*-tests). *C*,*D*, RL cells were pre-treated with the indicated concentrations of bortezomib (Bort, 100 nM), MG132 (10 μM) or DMSO for 1 h prior to treatment with soluble control antibody or anti-IgM for 6 h. Expression of PDCD4 and HSC70 was analyzed by immunoblotting. *C*, Representative results. *D*, Quantitation showing relative PDCD4 expression (mean ± SD) derived from 3 independent experiments with values for control antibody/DMSO-treated cells set to 1.0. The statistical significance of the differences is indicated (one-sample t-tests for comparisons to control, Student's t-tests for comparisons between samples).

Similar to CLL, BCR signaling also contributes to the pathogenesis of B-cell lymphoma, including the germinal center B-cell (GCB) and activated B-cell (ABC) subsets of DLBCL, and BL [24], [26]. Moreover, deletion of *Pdcd4* results in increased B-cell lymphomagenesis in mice [9]. We therefore extended our analysis to investigate potential regulation of PDCD4 downstream of the BCR in a small panel of B-cell lines, comprising RL (GCB-DLBCL), HBL-1 (ABC-DLBCL) and RAMOS (BL). In contrast to CLL cells, lymphoma cell lines express relatively high levels of sIgM and, consistent with previous studies [29], [42], [43], [44], soluble anti-IgM was used in these experiments since this is sufficient to induce strong signal responses in these cells. At 6 h, anti-IgM induced significant PDCD4 downregulation in each line (Fig. 5A,B), and in RL (Fig. 5C,D) and RAMOS cells (Supplementary Fig. 2) this downregulation was inhibited by MG132. Thus, similar to CLL cells, PDCD4 is down-regulated via proteasomal degradation in B-lymphoma cells following sIgM stimulation. PSI also modestly increased PDCD4 expression in RL cells in the absence of anti-IgM, indicating that PDCD4 may also be targeted for constitutive degradation in these cells (Fig. 5C,D).

**Fig. 6:**
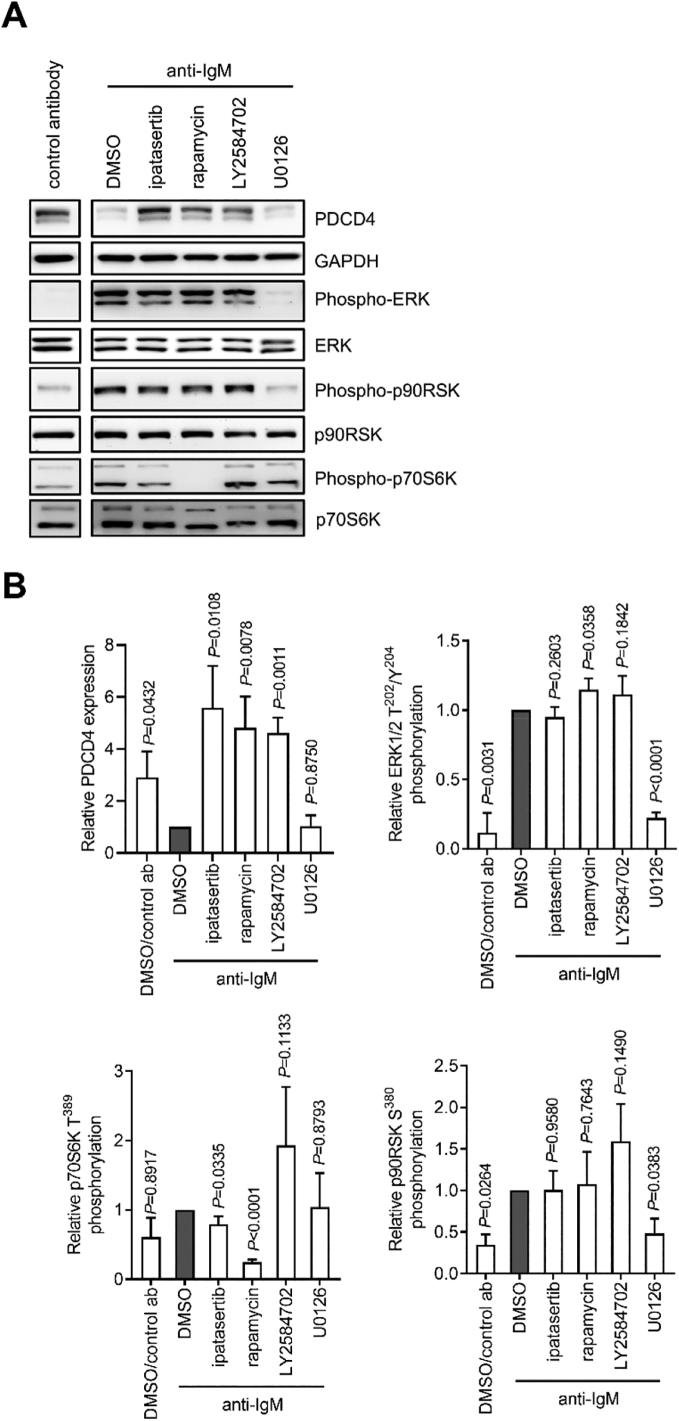
The effect of kinase inhibition on anti-IgM-induced PDCD4 down-regulation and signaling in RL cells. RL cells were pre-treated with ipatasertib, LY2584702, U0126 (all 10 μM), rapamycin (50 nM) or DMSO, or left untreated for 1 h prior to treatment with soluble control or anti-IgM antibodies for 6 h. Expression of PDCD4, GAPDH and total/phospho-p70S6K, ERK1/2 and p90RSK was analyzed by immunoblotting. *A*, Representative immunoblots. *B*, quantification (derived from 3 to 4 independent experiments). Graphs show mean (±SD) values with results for anti-IgM/DMSO-treated cells set to 1.0. The statistical significance compared to control cells is indicated (one-sample *t*-tests). Uncropped immunoblot images are shown in Supplementary Fig. 3.

We next investigated the effect of kinase inhibitors on anti-IgM-induced PDCD4 downregulation in RL cells (Fig. 6A,B). Interestingly, the pattern of response to specific kinase inhibitors differed between RL and CLL cells as, in contrast to CLL cells, AKT pathway inhibitors completely reversed anti-IgM-induced PDCD4 downregulation and U0126 had no effect in RL cells. Anti-IgM stimulation significantly increased phosphorylation of ERK1/2 and p90RSK, whereas changes in p70S6K were less dramatic as RL cells appeared to have relatively high levels of phosphorylation of this protein in the absence of stimulation. As expected, U0126 significantly reduced anti-IgM-induced ERK1/2 and p90RSK phosphorylation. However, in contrast to CLL cells, U0126 did not reduce p70S6K phosphorylation, indicating lack of inhibitory cross-talk in these cells. As expected, rapamycin (and to a lesser extent ipatasertib) reduced p70S6K. AKT pathway inhibitors also had no effect on ERK1/2 or p90RSK phosphorylation. Thus, RL cells differ from CLL due to the lack of MEK➔AKT pathway cross-talk and AKT pathway inhibition alone is able to fully reverse anti-IgM-induced PDCD4 down-regulation.

We also investigated the effect on AKT and MEK1/2 pathway inhibitors on basal PDCD4 expression in the absence of sIgM stimulation (ie, in control antibody-treated RL cells) (Supplementary Fig. 3A,B). AKT pathway inhibitors rapamycin and LY2584702 (but not ipatersertib, or U0126) significantly increased PDCD4 expression compared to DMSO-treated cells. Analysis of phosphorylation was more challenging in unstimulated cells due to the low levels detected for some markers. However, similar to anti-IgM-induced signaling in RL cells, it was notable that U0126 did reduce basal p90RSK, but not p70S6K phosphorylation.

These results demonstrate that both constitutive and anti-IgM-induced PDCD4 degradation in RL lymphoma cells appears to be predominantly mediated via the AKT pathway, with no clear contribution from the MEK pathway.

### 3.5 Combinations of kinase inhibitors fully reverse PDCD4 down-regulation and effectively suppress MYC induction in anti-IgM-treated CLL cells

**Fig. 7:**
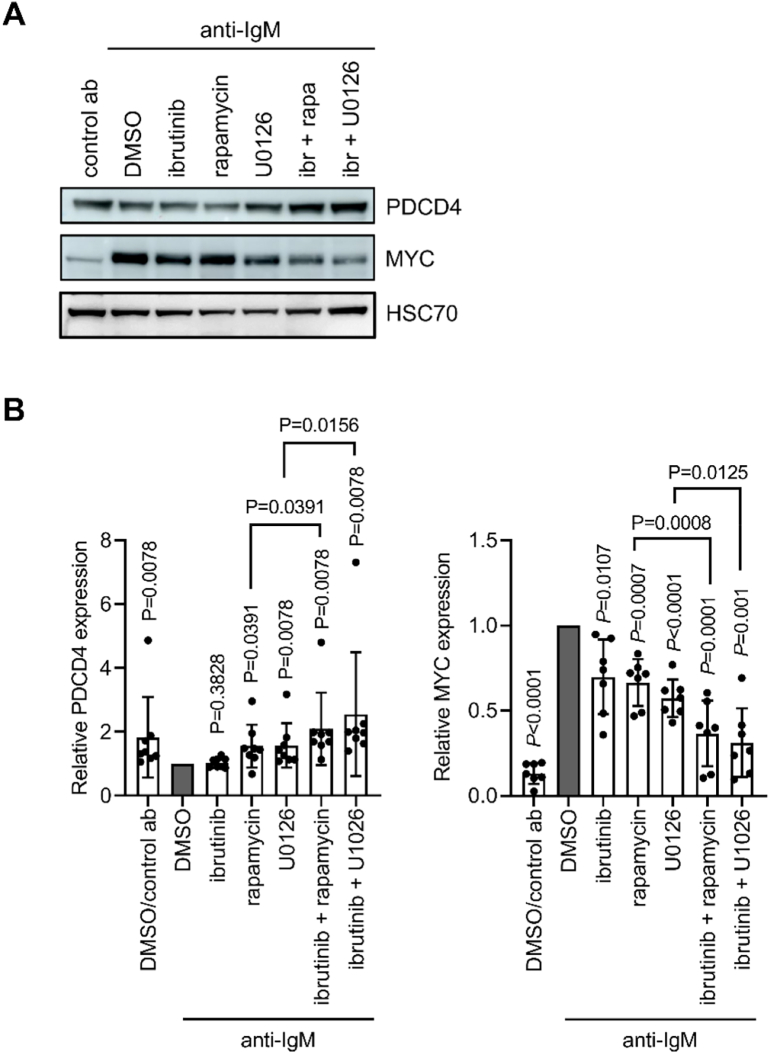
Effect of kinase inhibitor combinations on PDCD4 and MYC expression in anti-IgM stimulated CLL cells. CLL samples were pre-treated for 1 h with ibrutinib (ibr; 100 nM), rapamycin (50 nM), U0126 (10 μM), or DMSO, or the combination of ibrutinib and rapamycin or U0126 prior to treatment with control antibody or anti-IgM beads for 6 h. Expression of PDCD4, MYC and HSC70 was analyzed by immunoblotting. *A*, Representative immunoblots. *B*, Quantification showing results for individual samples and mean (±SD) expression with values for anti-IgM/DMSO-treated cells set to 1.0 (*n* = 8 for PDCD4 and *n* = 7 for MYC). The statistical significance of the indicated differences is indicated (Wilcoxon (PDCD4) and *t*-tests (MYC) (one sample for comparison to control or paired as indicated)).

In contrast to RL cells, single kinase inhibitors were not sufficient to fully reverse PDCD4 down-regulation in CLL cells in all samples (Fig. 4) and we therefore investigated the effects of combining either mTORC1 or MEK1/2 inhibition (using rapamycin or U0126, respectively) with the BTK inhibitor ibrutinib. Ibrutinib was selected as it is the main BCR-associated kinase inhibitor used for the treatment of CLL and was used at 100 nM as this concentration fully blocks BTK activity in cells and is clinically achievable [45]. Although BTK inhibition alone had no effect on PDCD4 downregulation, ibrutinib did increase the effects of rapamycin or U0126 such that PDCD4 down-regulation was completely reversed in cells treated with either combination (Fig. 7A,B). Investigation of single samples showed that, in contrast to single kinase inhibitors, dual inhibition was able to fully reverse PDCD4 downregulation even in samples with strong responses (Supplementary Fig. 4).

Since anti-IgM-induced MYC expression is dependent on eIF4A activity in CLL cells [23], [40], [41], we investigated the effects of combinatorial kinase inhibition on the expression of MYC. Individually, ibrutinib, rapamycin and U0126 only partially reversed anti-IgM-induced MYC expression (by ~30%; Fig. 7A,B). However, MYC induction was strongly repressed (by ~75%), in cells pre-treated with the combination of ibrutinib with rapamycin or U0126.

## 4 Discussion

Dysregulation of mRNA translation is a common feature in cancer, including lymphoma and leukemia, and targeted inhibition of translation is an exciting new area of drug discovery [46], [47]. We showed previously that activation of signaling downstream of sIgM in CLL cells resulted in down-regulation of PDCD4 (and up-regulation of its target for inhibition, eIF4A) [23]. Regulation of this PDCD4/eIF4Ai axis appears to be an important determinant of expression of MYC, a key oncoprotein for B-cell malignancies, since eIF4Ai effectively suppressed MYC induction following sIgM stimulation [34]. In this work, we investigated the signaling mechanisms that control PDCD4 expression downstream of the BCR, including the role of specific kinases.

PDCD4 can be regulated via multiple mechanisms, including transcriptional control and via the effects of miRNAs [10], [11], [12], [13]. However, in both CLL and B-lymphoma cells, anti-IgM-induced PDCD4 down-regulation was due to increased proteasomal degradation as it was reversed by PSI, including bortezomib which is used to treat multiple myeloma and mantle cell lymphoma. Although we have not identified the specific E3-ligase(s) that act on PDCD4 in CLL cells, candidates include SCF^ß-TRCP^ and IBTKα/CRL3 which have been shown to catalyze PDCD4 polyubiquitylation in other cell types [16], [17].

The upstream signaling pathways which mediate anti-IgM-induced PDCD4 down-regulation differed between CLL cells and B-lymphoma cells. Thus, in RL cells, down-regulation was tightly dependent on signaling via the AKT pathway with little involvement of the MEK pathway. In previous studies, PDCD4 degradation has shown to be linked to phosphorylation by AKT and/or p70S6K on serine residues in both the C- (Ser^67^, Ser^71^, Ser^76^) and N-terminal (Ser^457^) regions of the protein [3], [15], [17], [18], [21], [48]. However, in CLL cells, both the AKT and ERK pathways appeared to contribute to anti-IgM-induced down-regulation of PDCD4 expression. The ERK pathway may act on PDCD4 via three potential pathways. First, p90RSK can directly phosphorylate PDCD4 on Ser^76^ and Ser^457^, promoting 14–3-3 protein binding and degradation [19], [20]. Second, ERK1/2 can phosphorylate and inhibit TSC2, thereby relieving repression of mTORC1 (ie, cross-talk to the AKT pathway) and promoting p70S6K-mediated phosphorylation [49]. Third, ERK pathway signaling can enhance proteasome-mediated degradation of PDCD4 independent of effects on PDCD4 phosphorylation [15]. Interestingly, ERK➔AKT cross-talk appeared to operate in CLL cells, but not RL cells, as U0126 reduced phosphorylation of ERK1/2 and p90RSK, as well as p70S6K, and this may have contributed to the inability of single kinase inhibitors to fully reverse anti-IgM-induced PDCD4 down-regulation. Similar to other GCB-DLBCL, RL cells have inactivating mutations of *PTEN*, an inhibitor of the AKT pathway [50] and hyperactivation of AKT pathway in these cells may explain why signaling in these cells is heavily dependent on AKT.

It was interesting to note that AKT pathway inhibition also increased basal PDCD4 expression in RL cells (i.e. without sIgM stimulation), suggesting that PDCD4 is constitutively targeted for proteasomal degradation in these cells, even in the absence of anti-IgM stimulation. Consistent with this, PSI modestly increased PDCD4 expression in unstimulated cells. PDCD4 degradation in the absence of anti-IgM could be driven by serum factors (as in other cell types) [17], autocrine stimulation of other cell surface receptors or constitutive signaling emanating from the BCR. Constitutive BCR signaling has been described in various DLBCL cell lines, including HBL-1 cells analyzed here, and appears to be driven by engagement of the BCR by self-antigens expressed by the lymphoma cells or is a low-level, antigen-independent signal [29], [30], [31], [32], [33]. None of the kinases activated downstream of the BCR (including SYK and BTK) are specific for BCR signaling, so clarification of the role of constitutive BCR signaling in regulating PDCD4 degradation can only be definitively addressed by genetic manipulation of the BCR.

PDCD4 appears to be a determinant of MYC expression following BCR stimulation as inhibition of eIF4A effectively interferes with anti-IgM-induced MYC expression in CLL cells [34]. Consistent with this, combined inhibition of BTK with either rapamycin or U0126, which resulted in essentially full reversal of anti-IgM-induced PDCD4 down-regulation, was very effective at repressing MYC induction in CLL cells. Moreover, in PCs, sites of BCR stimulation and CLL cell proliferation in vivo [51], there was a striking inverse relationship between expression of MYC and PDCD4 where almost all MYC expressing cells lacked PDCD4 expression.

In contrast to RL cells, inhibition of neither the ERK nor AKT pathways alone was sufficient to consistently fully recover PDCD4 expression in anti-IgM-treated CLL cells, an effect that was most apparent in samples with the greatest degree of PDCD4 down-regulation. However, dual inhibition of BTK with either MEK1/2 or mTORC1 inhibition was much more effective at reversing PDCD4 down-regulation (and induction of MYC). Although ibrutinib (and other BTK inhibitors) can induce dramatic clinical responses in CLL patients, combinations of signaling inhibitors may be an effective strategy to boost responses and reduce the incidence of development of resistance and/or transformation to high grade disease [28]. Several studies have described the effects of multiple kinase inhibitors on CLL cells and have, for example, shown increased induction of apoptosis with dual inhibition of AKT and MEK [52], [53] although it should be noted that effective inhibition of AKT may be difficult to achieve clinically due to activation of compensatory mechanism [54]. However, this is the first study showing the efficacy of combining ibrutinib with either AKT or MEK pathway inhibition on regulators of mRNA translation and MYC expression.

## Author contributions

JT, MA-K, FS, FF, AJS, GP, AY conceived and designed the study; JT, SW, SM, K-RRB, RF, ARM, TB, MA-K, MC, FS, FF, AJS, GP and AY were involved in acquisition, analysis, or interpretation of data; JT, GP and AY drafted the work; all authors approved the submitted version of the manuscript.

## Funding

This work was supported by grants from the 10.13039/501100000265Medical Research Council, 10.13039/501100000289Cancer Research UK (C2750/A23669, C42023/A29370) and the Southampton Experimental Cancer Medicine and Cancer Research Centres (C24563/A15581, C34999/A18087).

## Declaration of Competing Interest

The authors report no relevant conflicts-of-interest.

## Appendix A Supplementary data

Supplementary material Supplementary material: related to the main article Image 1
