# Supplementary material for: B-cell receptor signaling induces proteasomal degradation of PDCD4 via MEK1/2 and mTORC1 in malignant B cells
Source: Cell Signal. 2022 Jun;94:110311. doi: 10.1016/j.cellsig.2022.110311 (PMC9077442; doi:10.1016/j.cellsig.2022.110311)
Supplement: Supplementary material — related to the main article [file mmc1.pptx]

## Slide 1
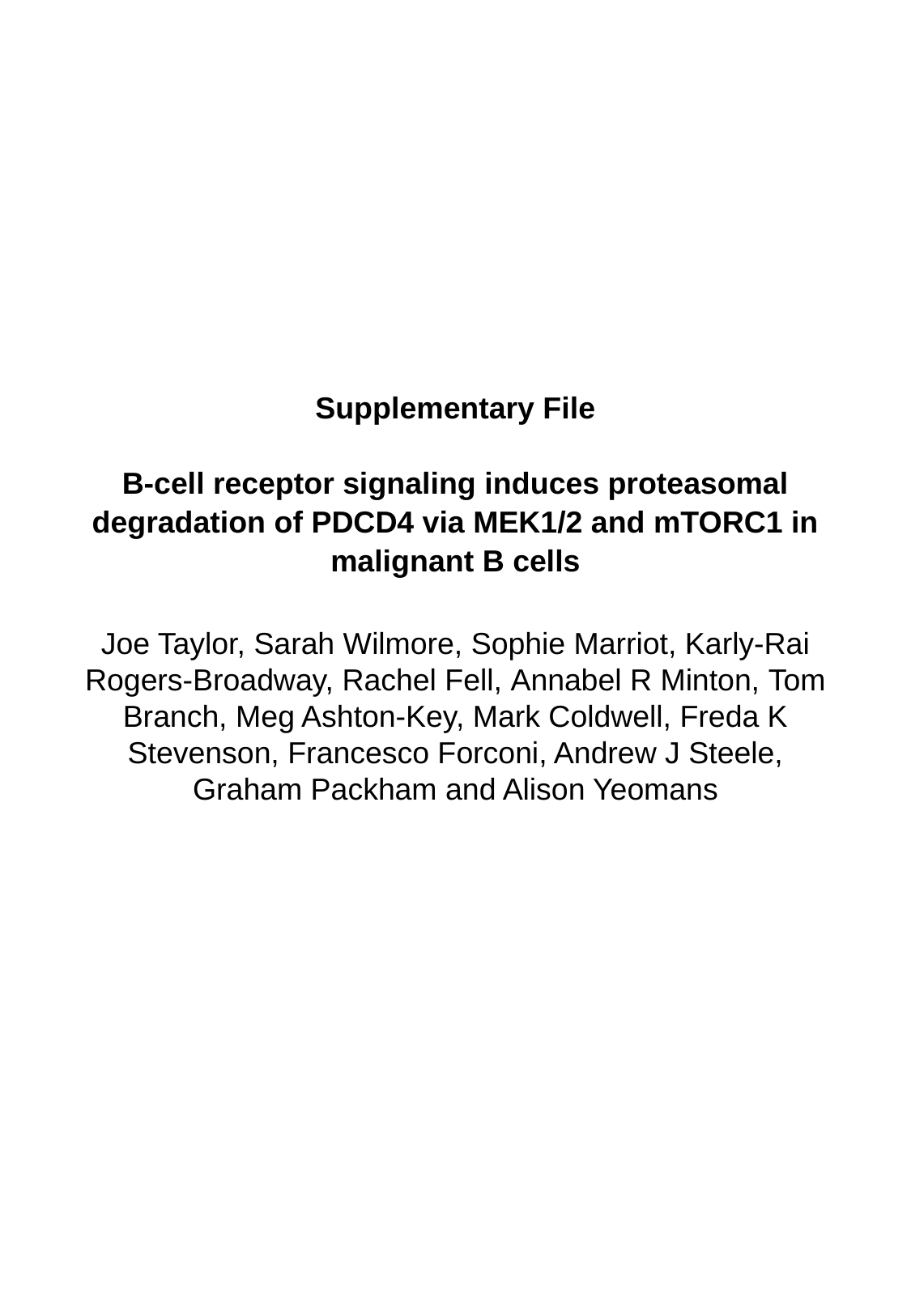

Supplementary File
B-cell receptor signaling induces proteasomal degradation of PDCD4 via MEK1/2 and mTORC1 in malignant B cells
Joe Taylor, Sarah Wilmore, Sophie Marriot, Karly-Rai Rogers-Broadway, Rachel Fell, Annabel R Minton, Tom Branch, Meg Ashton-Key, Mark Coldwell, Freda K Stevenson, Francesco Forconi, Andrew J Steele, Graham Packham and Alison Yeomans

## Slide 2
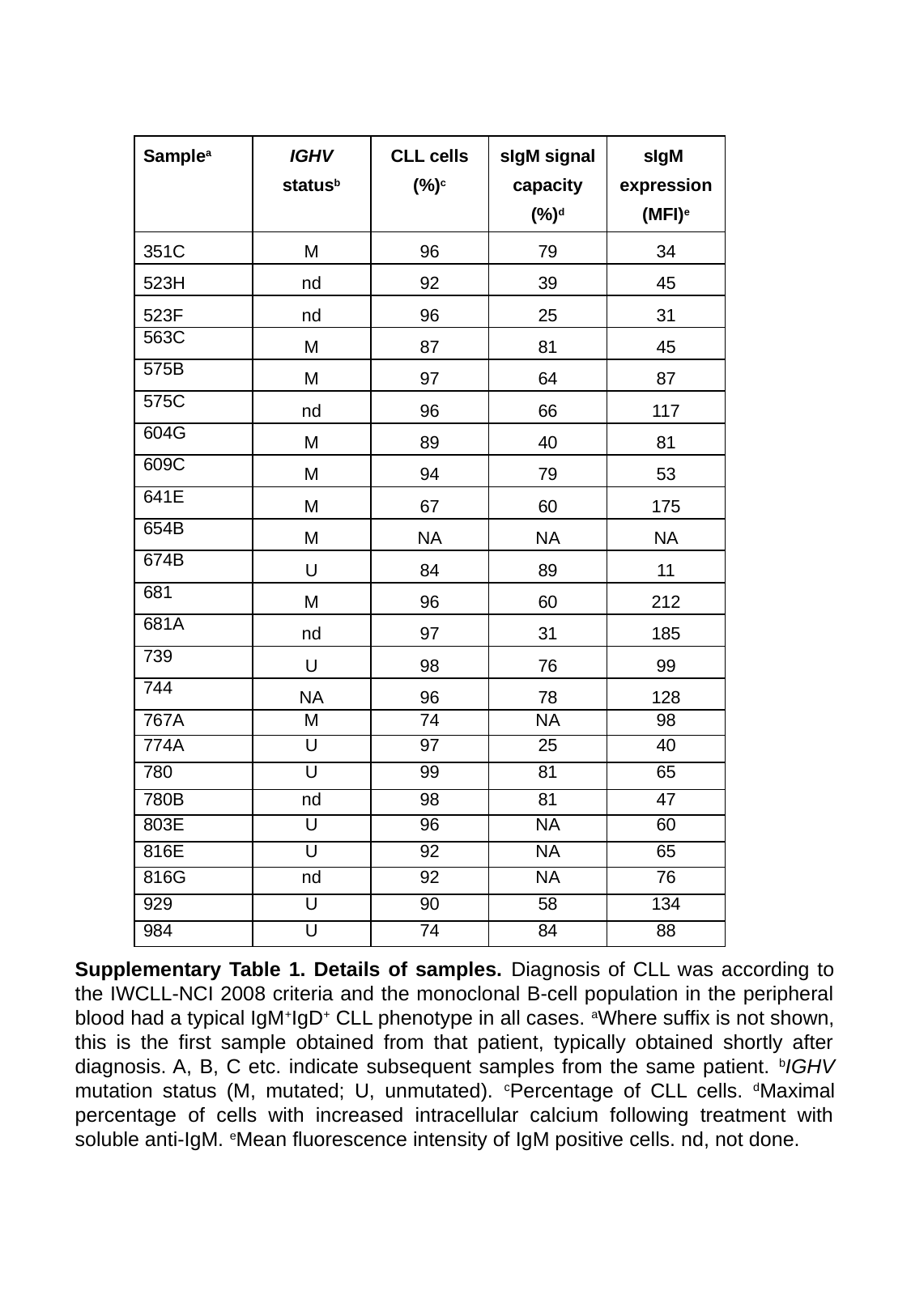

| Samplea | IGHV statusb | CLL cells (%)c | sIgM signal capacity (%)d | sIgM expression (MFI)e |
| --- | --- | --- | --- | --- |
| 351C | M | 96 | 79 | 34 |
| 523H | nd | 92 | 39 | 45 |
| 523F | nd | 96 | 25 | 31 |
| 563C | M | 87 | 81 | 45 |
| 575B | M | 97 | 64 | 87 |
| 575C | nd | 96 | 66 | 117 |
| 604G | M | 89 | 40 | 81 |
| 609C | M | 94 | 79 | 53 |
| 641E | M | 67 | 60 | 175 |
| 654B | M | NA | NA | NA |
| 674B | U | 84 | 89 | 11 |
| 681 | M | 96 | 60 | 212 |
| 681A | nd | 97 | 31 | 185 |
| 739 | U | 98 | 76 | 99 |
| 744 | NA | 96 | 78 | 128 |
| 767A | M | 74 | NA | 98 |
| 774A | U | 97 | 25 | 40 |
| 780 | U | 99 | 81 | 65 |
| 780B | nd | 98 | 81 | 47 |
| 803E | U | 96 | NA | 60 |
| 816E | U | 92 | NA | 65 |
| 816G | nd | 92 | NA | 76 |
| 929 | U | 90 | 58 | 134 |
| 984 | U | 74 | 84 | 88 |
Supplementary Table 1. Details of samples. Diagnosis of CLL was according to the IWCLL-NCI 2008 criteria and the monoclonal B-cell population in the peripheral blood had a typical IgM+IgD+ CLL phenotype in all cases. aWhere suffix is not shown, this is the first sample obtained from that patient, typically obtained shortly after diagnosis. A, B, C etc. indicate subsequent samples from the same patient. bIGHV mutation status (M, mutated; U, unmutated). cPercentage of CLL cells. dMaximal percentage of cells with increased intracellular calcium following treatment with soluble anti-IgM. eMean fluorescence intensity of IgM positive cells. nd, not done.

## Slide 3
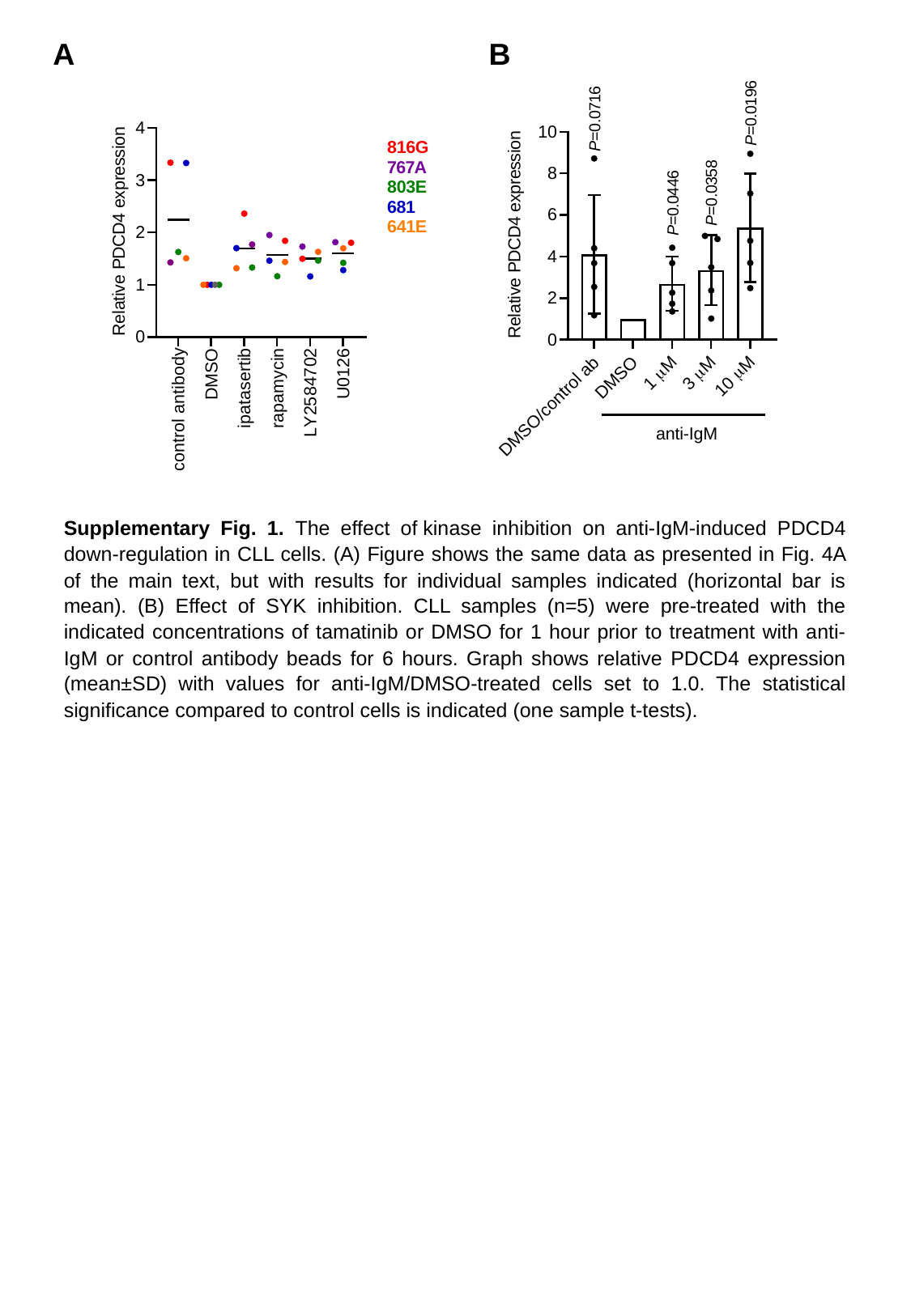

A
B
Supplementary Fig. 1. The effect of kinase inhibition on anti-IgM-induced PDCD4 down-regulation in CLL cells. (A) Figure shows the same data as presented in Fig. 4A of the main text, but with results for individual samples indicated (horizontal bar is mean). (B) Effect of SYK inhibition. CLL samples (n=5) were pre-treated with the indicated concentrations of tamatinib or DMSO for 1 hour prior to treatment with anti-IgM or control antibody beads for 6 hours. Graph shows relative PDCD4 expression (mean±SD) with values for anti-IgM/DMSO-treated cells set to 1.0. The statistical significance compared to control cells is indicated (one sample t-tests).

## Slide 4
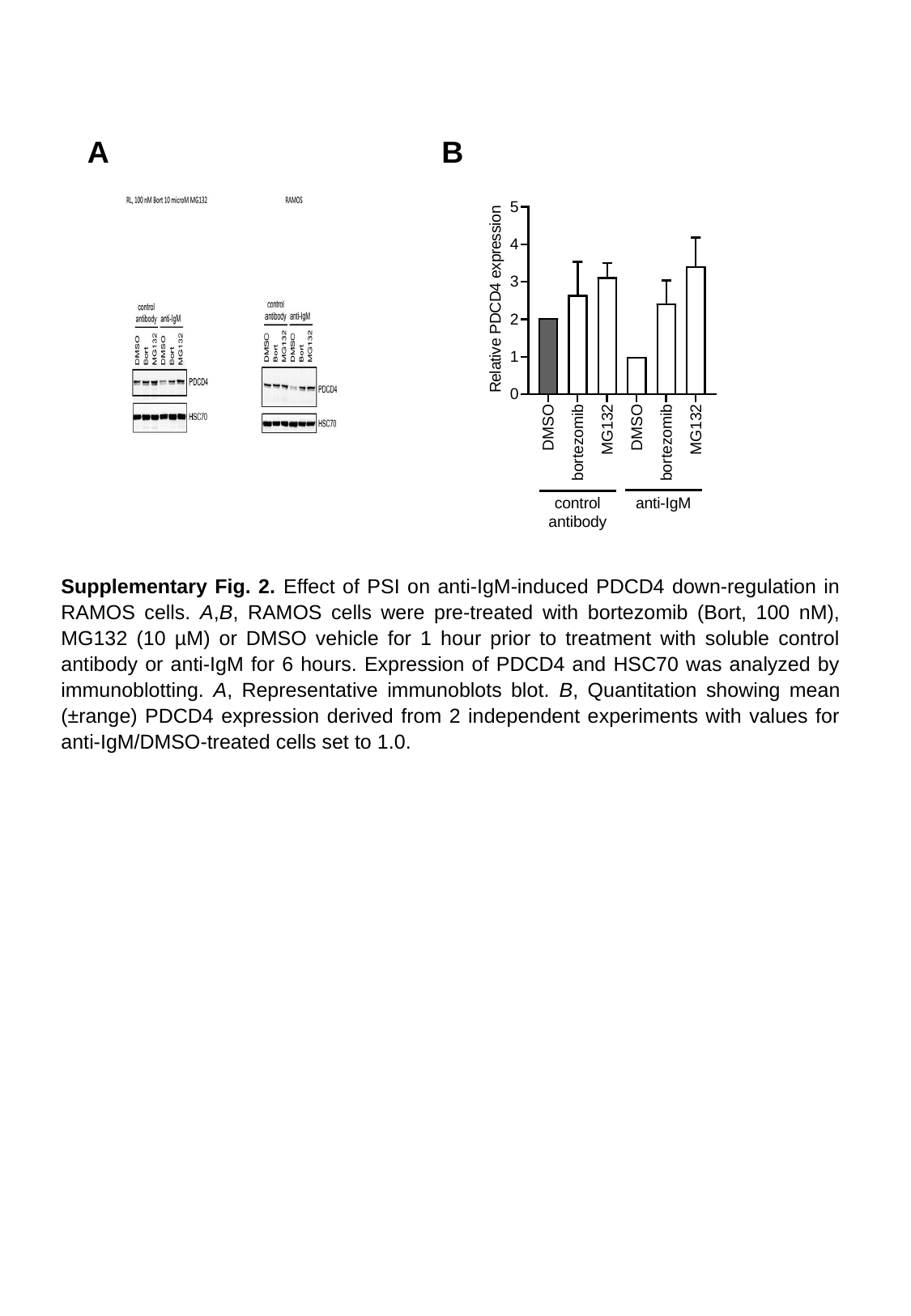

A
B
Supplementary Fig. 2. Effect of PSI on anti-IgM-induced PDCD4 down-regulation in RAMOS cells. A,B, RAMOS cells were pre-treated with bortezomib (Bort, 100 nM), MG132 (10 µM) or DMSO vehicle for 1 hour prior to treatment with soluble control antibody or anti-IgM for 6 hours. Expression of PDCD4 and HSC70 was analyzed by immunoblotting. A, Representative immunoblots blot. B, Quantitation showing mean (±range) PDCD4 expression derived from 2 independent experiments with values for anti-IgM/DMSO-treated cells set to 1.0.

## Slide 5
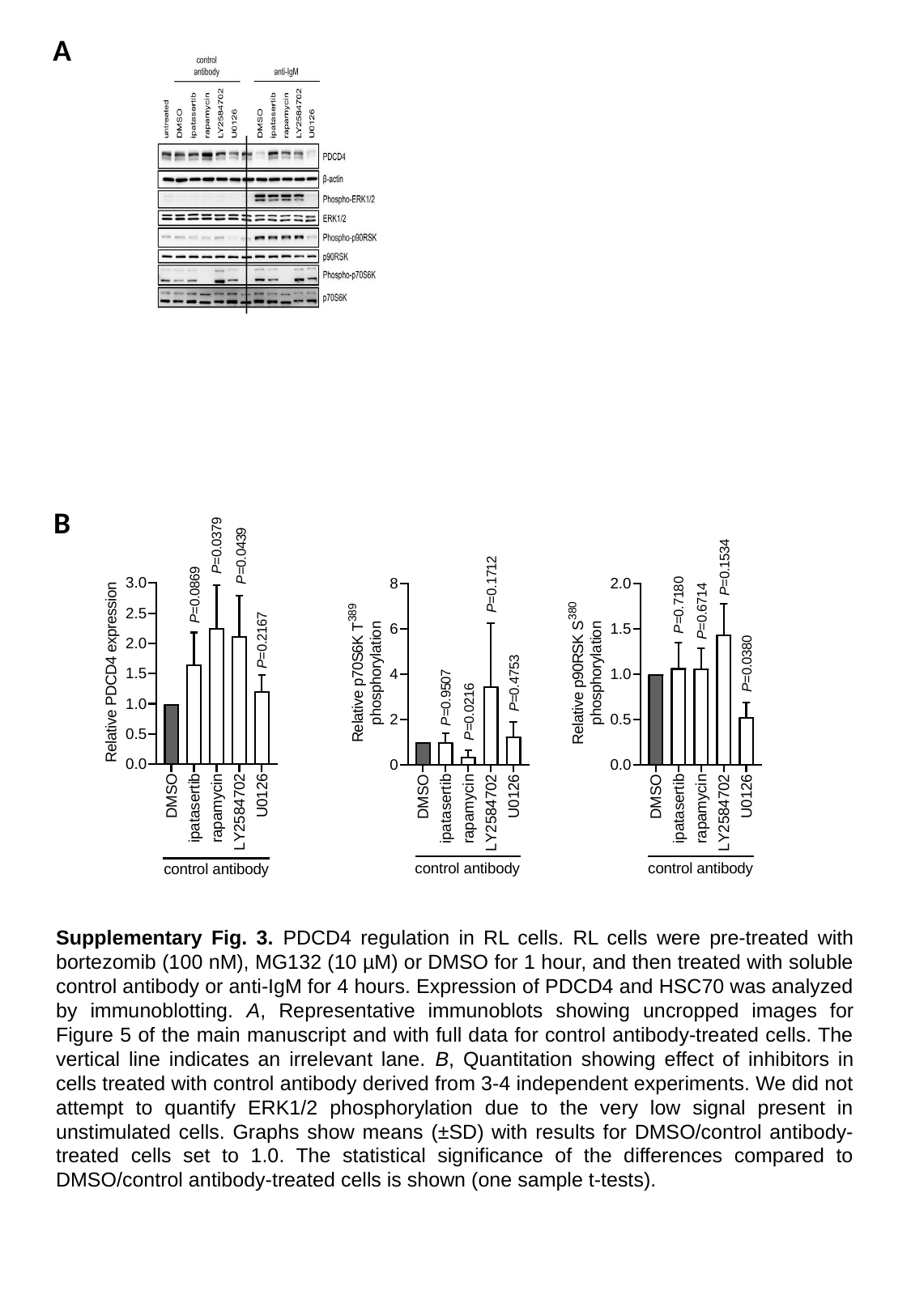

A
B
Supplementary Fig. 3. PDCD4 regulation in RL cells. RL cells were pre-treated with bortezomib (100 nM), MG132 (10 µM) or DMSO for 1 hour, and then treated with soluble control antibody or anti-IgM for 4 hours. Expression of PDCD4 and HSC70 was analyzed by immunoblotting. A, Representative immunoblots showing uncropped images for Figure 5 of the main manuscript and with full data for control antibody-treated cells. The vertical line indicates an irrelevant lane. B, Quantitation showing effect of inhibitors in cells treated with control antibody derived from 3-4 independent experiments. We did not attempt to quantify ERK1/2 phosphorylation due to the very low signal present in unstimulated cells. Graphs show means (±SD) with results for DMSO/control antibody-treated cells set to 1.0. The statistical significance of the differences compared to DMSO/control antibody-treated cells is shown (one sample t-tests).

## Slide 6
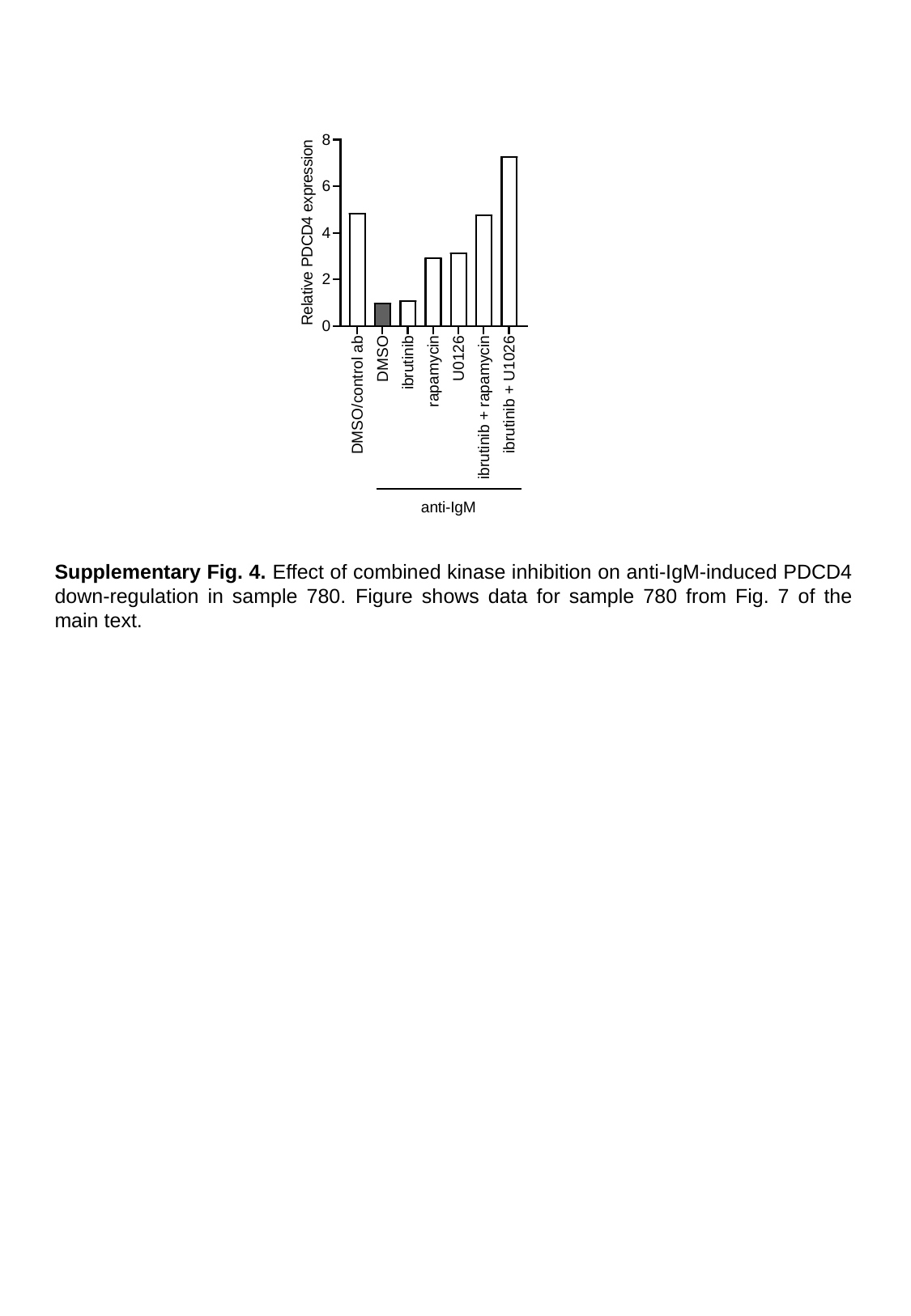

Supplementary Fig. 4. Effect of combined kinase inhibition on anti-IgM-induced PDCD4 down-regulation in sample 780. Figure shows data for sample 780 from Fig. 7 of the main text.
